# Tim-3: An Activation Marker and Activation Limiter of Innate Immune Cells

**DOI:** 10.3389/fimmu.2013.00449

**Published:** 2013-12-10

**Authors:** Gencheng Han, Guojiang Chen, Beifen Shen, Yan Li

**Affiliations:** ^1^Department of Immunology, Beijing Institute of Basic Medical Sciences, Beijing, China

**Keywords:** TIM-3, innate immunity, negative regulation, tolerance, homeostasis

## Abstract

Tim-3 was initially identified on activated Th1, Th17, and Tc1 cells and induces T cell death or exhaustion after binding to its ligand, Gal-9. The observed relationship between dysregulated Tim-3 expression on T cells and the progression of many clinical diseases has identified this molecule as an important target for intervention in adaptive immunity. Recent data have shown that it also plays critical roles in regulating the activities of macrophages, monocytes, dendritic cells, mast cells, natural killer cells, and endothelial cells. Although the underlying mechanisms remain unclear, dysregulation of Tim-3 expression on these innate immune cells leads to an excessive or inhibited inflammatory response and subsequent autoimmune damage or viral or tumor evasion. In this review, we focus on the expression and function of Tim-3 on innate immune cells and discuss (1) how Tim-3 is expressed and regulated on different innate immune cells; (2) how it affects the activity of different innate immune cells; and (3) how dysregulated Tim-3 expression on innate immune cells affects adaptive immunity and disease progression. Tim-3 is involved in the optimal activation of innate immune cells through its varied expression. A better understanding of the physiopathological role of the Tim-3 pathway in innate immunity will shed new light on the pathogenesis of clinical diseases, such as autoimmune diseases, chronic viral infections, and cancer, and suggest new approaches to intervention.

## Introduction

T cell immunoglobulin domain and mucin domain (Tim)-3, initially identified on terminally differentiated Th1 cells, negatively regulates the T cell response by inducing T cell apoptosis (1). Tim-3 was later found to be also present on activated Th17 and Tc1 cells (2–4), and many studies have shown that dysregulation of Tim-3 expression on CD4^+^ T cells and CD8^+^ T cells is closely related to many autoimmune diseases, viral infections, and cancer [e.g., (2–4)]. For example, Liberal et al. (2) showed that reduced levels of Tim-3 on CD4^+^ T effector cells result in enhanced T cell activation in patients with autoimmune hepatitis. In contrast, Wu et al. (3) showed that upregulation of Tim-3 on CD8^+^ T cells leads to T cell paralysis in patients with HIV infection.

Recently, Tim-3 was also shown to play important roles on innate immune cells, including macrophages/monocytes (5–9), dendritic cells (DCs) (10–13), and natural killer (NK) cells (14–16). Excessive upregulation of Tim-3 expression on these innate immune cells was found to lead to exacerbation of clinical diseases (3, 4, 11, 16). In contrast to the relatively clear mechanisms of Tim-3-mediated T cell death or exhaustion (1–4), the mechanisms by which Tim-3 regulates the activities of innate immune cells remain largely unclear. Here, we will focus on the function of Tim-3 in some important innate immune cells and how it contributes to immune homeostasis by regulating their activity.

### Tim-3 is actively involved in regulating macrophage/monocyte activity

Macrophages play critical roles in inflammatory responses, and their dysregulation is associated with excessive or uncontrolled inflammation. Many regulatory mechanisms exist to maintain macrophage homeostasis. Recently, Tim-3, a negative immune regulator of effector T cells, was shown to be involved in the regulation of macrophage activity (5–7). Monney et al. (5) reported that the *in vivo* administration of anti-Tim-3 antibody increased the number and activation level of macrophages and enhanced the clinical and pathological severity of experimental autoimmune encephalomyelitis. Although the underlying mechanisms were not clearly determined, their data indicated that Tim-3 may negatively regulate macrophage activation and/or function and thus affect the progression of autoimmune diseases.

Subsequently, Frisancho-Kiss et al. (6) showed that viral infection of mice led to rapid Tim-3 expression on macrophages in the peritoneum, spleen, and heart and that blockade of the Tim-3 pathway led to decreased CD80 expression on macrophages and an enhanced inflammatory response. These findings suggest that Tim-3 is involved in the earlier stages of an immune response and may act as an inhibitor of macrophage activation. Recent data from our own laboratory shed new light on the roles of Tim-3 in macrophage activation by showing that Tim-3 expression on macrophages was unregulated in sepsis or in response to lipopolysaccharide (LPS) stimulation (7), suggesting that Tim-3 acts as an activation marker. Consistent with the data of Frisancho-Kiss et al. (6), we also showed that blockade of the Tim-3 pathway during sepsis *in vivo* or downregulation of Tim-3 on macrophages *in vitro* led to enhanced macrophage activation (7). Our findings show that Tim-3 can be upregulated on macrophages in response to stimuli and that it may also act to suppress macrophage activity.

As regards monocytes, Ma et al. (8, 9, 17) showed that Tim-3 is constitutively expressed on resting human CD14^+^ monocytes/macrophages and that it acts to limit IL-12 production. Their studies demonstrated that stimulation with the Toll-like receptor 4 (TLR4) ligand LPS or the TLR7/8 ligand R848 downregulates Tim-3 expression, leading to enhanced IL-12 expression, consistent with our previous data in macrophages, which showed that LPS downregulates the expression of Tim-3 in a dose- and time-dependent manner, leading to enhanced TNF-α and IL-6 production (7). These data suggest that Tim-3 is dynamically expressed on monocytes/macrophages and that its expression is closely related to the activity of these cells. However, in patients with chronic hepatitis C virus (HCV) infection, Tim-3 is overexpressed on unstimulated and TLR-stimulated monocytes and macrophages and this is associated with decreased IL-12 expression compared to healthy subjects (8). A previous report showed that TLR ligand stimulation (malaria parasites) resulted in decreased Tim-3 expression on monocytes from HIV (−) controls, but not those from HIV (+) donors (18). Thus, increased Tim-3 expression on monocytes may act to suppress their activity, whereas reduced Tim-3 expression may be associated with monocyte activation. In such a scenario, increased Tim-3 expression and a failure in the downregulation of Tim-3 on macrophages/monocytes by factors such as LPS and TLR4 may act as markers for dysfunctional macrophages/monocytes, as demonstrated previously for CD4^+^ and CD8^+^ T cells (2, 3).

In addition to the role of Tim-3 as a negative regulator of macrophage activation, blockade of the Tim-3 pathway has been shown to inhibit the phagocytic potential of uterine macrophages, resulting in a buildup of apoptotic bodies at the uteroplacental interface that elicits a local immune response (19), suggesting that Tim-3 regulates phagocytosis by macrophages. In addition, a recent study (20) demonstrated that Tim-3 recognizes apoptotic cells through the FG loop in the IgV domain and is crucial for the clearance of apoptotic cells by phagocytes. This study also demonstrated reduced cross-presentation of dying cell-associated antigens following Tim-3 pathway blockade *in vitro* and *in vivo*. These data suggest that Tim-3 contributes to peripheral tolerance by regulating macrophage-mediated phagocytosis and antigen cross-presentation. However, it is still unclear how dysregulated Tim-3 expression affects phagocytosis and antigen cross-presentation and subsequent disease progression.

Dynamic expression of Tim-3 on macrophages is also observed in autoimmune diseases and is associated with the polarization of macrophages. Working with an experimental autoimmune neuritis (EAN) model, Zhang et al. (21) showed that, during the recovery phase, Tim-3 expression is increased on CD68^+^ and CD163^+^ macrophages and that recovery from EAN is accompanied by increased Tim-3 expression on CD163^+^ cells. In response to cytokines, resident macrophages, or recruited monocytes differentiate into two distinct subsets referred to as classically activated (M1) or alternatively activated (M2) macrophages (22, 23). M1 macrophages primarily function to promote inflammation and tissue damage, whereas M2 macrophages play a major role in damping down the inflammatory response. As CD163 expression is upregulated by anti-inflammatory signals (24), high CD163 expression is considered to be a marker for M2 macrophages (25). The data reported by Zhang et al. (21) suggest that Tim-3 expression is associated with macrophage polarization and that enhanced Tim-3 expression on CD163^+^ macrophages (M2) contributes to immune regulation by limiting macrophage activation. In agreement with this, Frisancho-Kiss et al. (26) demonstrated that Tim-3 was upregulated on M2 cells in gonadectomized male mice and mediated an anti-inflammatory response. These data again show that Tim-3 is kinetically expressed on macrophages and that it dynamically regulates their activity.

**Figure 1:**
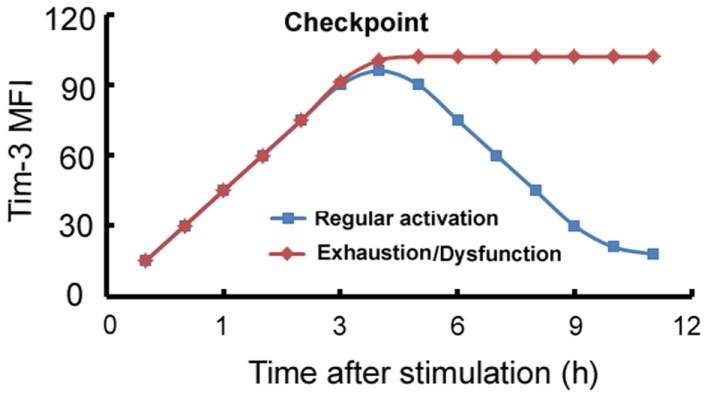
**Model of the Tim-3-mediated regulation of innate immune responses**. On innate immune cells, Tim-3 can be either constitutively expressed at a certain level which we term “checkpoint,” or its expression can be induced to this level, at which it acts as an activation limiter or “brake.” When Tim-3 can be normally downregulated, its “limiter” or “brake” function is suppressed and the cells become functional; we term this process “regular activation.” However, in circumstances, such as chronic inflammation, in which Tim-3 is overexpressed or cannot be downregulated, Tim-3 signaling maintains the cell in a quiescent state, referred to as “exhaustion” or “dysfunction.” *x*-axis: stimulation time (h); *y*-axis: mean fluorescence intensity (MFI) of Tim-3 on macrophages.

A model can be developed summarizing the expression and function of Tim-3 on macrophages/monocytes. Tim-3 is constitutively expressed on innate immune cells at a certain level or its expression can be induced to this level, which we will call “checkpoint” at which it acts as an activation limiter or “brake.” In circumstances in which Tim-3 can be normally downregulated, its “limiter” or “brake” function is suppressed and the cells become functional; we will call this process “regular activation.” However, in circumstances, such as chronic inflammation, in which Tim-3 is overexpressed or its expression can no longer be downregulated by factors such as LPS, Tim-3 signaling maintains the cell in a quiescent state, referred to as “exhaustion” or “dysfunction” (Figure 1).

Although the above data allow us to propose a functional model of the involvement of Tim-3 in the regulation of immune cell activity, three points remain to be determined. First, the factors that regulate Tim-3 expression in different conditions must be identified. A partial answer has been provided by data showing that TLR signaling is involved in the regulation of Tim-3 on some immune cells (7–9). For example, our previous study (7) showed that LPS (a TLR4 ligand) can bidirectionally regulate Tim-3 expression on macrophages, while other data (9) showed that LPS and R848 (a TLR7/8 ligand) can downregulate Tim-3 expression on monocytes, which is upregulated in HCV infection (8, 9). Other factors that contribute to the dynamic expression of Tim-3 on macrophages/monocytes remain to be identified. Second, the mechanisms by which Tim-3 regulates macrophage/monocyte activity are also unclear. As shown in our previous study (7), Tim-3 signaling increases the LPS-induced phosphorylation of PI3K-AKT and also the expression of A20, which inhibits NF-κB activation and subsequent macrophage activation, but the detailed mechanisms remain to be determined. Third, the mechanisms by which dysregulated Tim-3 expression on macrophages/monocytes affects adaptive immunity are not known. Tim-3 expression has been shown to be associated with the polarization of M1 and M2 cells. Moreover, M1 polarization is associated with Th1 polarization, and M2 polarization is associated with Th2 polarization (22, 23). Wang et al. (27) showed that, in HCV-infected patients, Tim-3 overexpression on monocytes is positively correlated with IL-23 levels and negatively correlated with IL-12 levels, leading to a Th17-biased response. These data suggest that Tim-3 also contributes to the homeostasis of adaptive immunity by affecting the polarization and/or activity of innate immune cells. However, the underlying mechanisms remain unclear, including, for example, how Tim-3 expression affects the polarization and activity of macrophages/monocytes, leading to biased T cell polarization.

### The roles of Tim-3 on dendritic cells are context-dependent

Recently, Chiba et al. (10) reported that tumor-infiltrating DCs show increased Tim-3 expression and suppress nucleic-acid-mediated innate immune responses as a result of the interaction between Tim-3 and the alarmin HMGB1, and that, when Tim-3 signaling was blocked using anti-Tim-3 monoclonal antibody, chemotherapy was found to be more effective, suggesting that Tim-3 plays a role in tumor pathogenesis. They also reported that, after stimulation of bone marrow derived DCs (BMDCs) with nucleic-acid TLR agonists, expression of the cytokines interferon-β1 (IFN-β1), IFN-α4, IL-6, and IL-12 was much lower in BMDCs from wild-type mice (Tim-3^+^) than in those from Tim-3-deficient mice (Tim-3^−^). Their findings suggest that Tim-3 signaling negatively regulates the nucleic-acid-mediated anti-tumor immune response of DCs (11). However, early data indicated that Tim-3 positively regulates DC activities. For example, Anderson et al. (12) reported that Tim-3 is constitutively expressed on DCs and that activation of Tim-3 signaling by Gal-9 or by an agonist Tim-3 antibody promotes LPS-induced DC activation. These findings suggest a model in which Tim-3 promotes innate immunity (promotion). Consistent with this report, Nagahara et al. (13) showed that, in Meth-A tumor-bearing mice, Gal-9 increased the number of Tim-3^+^ CD86^+^ mature DCs *in vivo* and prolonged survival, and that depletion of Tim-3^+^ DCs from Gal-9-treated tumor-bearing mice decreased anti-tumor immunity, indicating that Tim-3 expression on DCs plays a critical role in Gal-9-promoted anti-tumor immunity. In addition, Kanzaki et al. (28) demonstrated that Gal-9-Tim-3 signaling induced TNF-α production in cultured DCs in a dose-dependent manner. Together, these data suggest that Tim-3 plays a dual role in regulating the activity of DCs.

However, other data do not support the idea that Tim-3 signaling promotes the activity of DCs. If Tim-3 is a negative regulator of the activation of T cells (1–4), macrophages (6, 7), and monocytes (9), it is not clear why it would be a positive regulator of DC activity (12, 13). Additionally, although Gal-9 induces DC activation, it does not necessarily do so by binding to Tim-3, as it can bind to receptor(s) other than Tim-3 (29, 30). For example, Kadowaki et al. (29) showed that Gal-9 increased the frequency of CD11c (high) plasmacytoid DC-like macrophages *in vitro* in a Tim-3-independent manner and prolonged the survival of lung cancer-bearing mice. In addition, although previous reports suggested that Tim-3 negatively regulates murine T cell responses (4) and is upregulated on exhausted murine and human T cells, the function of which can be restored by anti-Tim-3 antibodies (3, 4), Leitner et al. (30) recently reported that the activation of human T cells is not affected by anti-Tim-3 antibodies and that Tim-3 on human T cells does not act as a receptor for Gal-9.

More investigations on the Tim-3 signaling pathway are needed to clearly define the exact roles of Tim-3 in DCs. As a different level of Tim-3 expression is seen on *in vitro* cultured DCs (28) and tumor-derived DCs (10), the roles of Tim-3 in DCs might be context-dependent. It is worth noting that Tim-3 overexpression negatively regulates DC activity (10, 11), indicating that dysregulated Tim-3 expression on DCs may also act as a marker of DC dysfunction, as in to monocytes (8, 10, 18).

### Tim-3 stimulates mast cells to produce Th2 cytokines and regulates the Th1/Th2 balance *in vivo*

It is known that Tim-3 can negatively regulate the Th1 response by inducing Th1 cell apoptosis (1). However, it has also been found to regulate the Th2 cytokine response in experimental asthma. Li et al. (31) showed that Tim-3 stimulated mast cells produced Th2 cytokines and that anti-Tim-3 antibodies reduced asthmatic inflammation. In agreement with this, Kearley et al. (32) found that administration of anti-Tim-3 monoclonal antibodies before each airway challenge in mice resulted in significantly reduced airway hyperreactivity, a decreased Th2 response, and an increased Th1 response in the lung. Nakae et al. (33) reported that Tim-3 was constitutively expressed on mast cells and that, after IgE and antigen stimulation, its expression was upregulated and enhanced antigen-dependent Th2 cytokine secretion. The observations of these three groups suggested that Tim-3 may influence T cell-mediated immune responses, in part, by effects on mast cells. Experiments were then performed to identify factor(s) that might regulate Tim-3 expression on mast cells. Kim et al. (34) found that Tim-3 mRNA levels were increased in the human mast cell line HMC-1 following stimulation with TGF-β1, but not with IFN-α, IFN-γ, TNF-α, or IL-10, and that the region −349 to +144 base pairs relative to the transcription start site of the Tim-3 promoter was crucial for basal and TGF-β1-induced Tim-3 promoter activity in these cells. In addition, Yoon et al. (35) reported that the mitogen-activated protein kinase Erk kinase was involved in the upregulation of Tim-3 transcription in Jurkat cells (a human T cell line) stimulated with phorbol-12-myristate-13-acetate and in HMC-1 cells stimulated with TGF-β. Frisancho-Kiss et al. (6) also demonstrated that blockade of the Tim-3 pathway led to decreased CD80 expression on mast cells and an enhanced inflammatory response, showing that Tim-3 actively alters the phenotype and activity of mast cells.

Together, these data suggest that, by increasing the Th2 response while inhibiting the Th1 cytokine-induced inflammatory response, Tim-3 signaling in mast cells influences the balance of immune responses *in vivo*. However, the mechanism by which the Tim-3 signaling pathway promotes Th2 cytokine production in mast cells remains unclear.

### Tim-3 acts as an NK cell maturation marker and is necessary for their cytotoxicity, but its expression is dysregulated during chronic viral infection

Recent data have shown that the activity of NK cells can also be regulated by Tim-3 (14– 16). NK cells are involved in innate immunity and play an important protective role against viral infection and cancer. This effect is achieved through a complex mosaic of inhibitory and activating receptors expressed by NK cells that ultimately determines the magnitude of the NK cell response. Ndhlovu et al. (14) showed that many cytokines, such as IFN-α and combinations of IL-12+IL-18 or IL-12+IL-15, could induce Tim-3 upregulation on NK cells. Furthermore, NK cell IFN-γ production and cytotoxic ability were associated with the NK cells with the highest Tim-3 levels and these authors therefore concluded that Tim-3 might act as a marker of fully mature and functional NK cells. In agreement with these findings, Gleason et al. (15) demonstrated that Tim-3 was upregulated on human NK cells after activation and promoted IFN-γ production in response to Gal-9. However, Ndhlovu et al. (14) demonstrated that, when Tim-3 was cross-linked on NK cells with antibodies or encountered target cells that expressed its ligand Gal-9, it suppressed NK cell-mediated cytotoxicity, showing that Tim-3 also suppresses NK cell functions. A study by Ju et al. (16) also supports a negative regulatory role of Tim-3 on the activity of NK cells, as they showed that chronic hepatitis B infection upregulated Tim-3 expression on NK cells and subsequently suppressed NK cell function and that this process was reversed by blockade of the Tim-3 pathway. These data suggest that Tim-3 has opposing effects on NK cells. Although Tim-3 may be a marker of NK cell activation, its overexpression may also restrain NK cell function in certain situations (14, 16).

Recent data have also indicated that Tim-3 expression on NK cells is dysregulated during chronic viral infection. For example, Finney et al. (18) reported that Tim-3 was overexpressed on NK cells isolated from patients with chronic untreated HIV infection and that these cells were significantly less responsive to TLR stimulation than cells from HIV(−) controls and that 6 months of combination antiretroviral therapy resulted in partial restoration of both Tim-3 levels and NK cell activation. These data show that chronic HIV infection leads to increased Tim-3 expression on NK cells and that this increased expression is associated with a dysfunction phenotype of NK cells. However, how, or to what extent, the compromised responses of NK cells in chronic virus infections, such as hepatitis B (16) and HIV(18, 36) infection, relate to Tim-3 expression requires further investigation. If Tim-3 is also a marker of innate immune cell dysfunction in addition to being a marker of CD4+ and CD8+ T cell dysfunction (2–4), dysregulated Tim-3 expression may be associated with a more profound immune deficit of both adaptive and innate immune cells.

### Tim-3 expression is upregulated on the endothelium in humans or mice with lymphoma and acts as an inhibitor of CD4^+^ T cells

Wu et al. (37) showed that, in mice, Tim-3 is also expressed on vascular endothelial cells (ECs), but does not function as a Gal-9 receptor on these cells, and, instead, mediates the interaction of ECs with tumor cells or T cells. For example, Tim-3 on ECs, by binding to a putative receptor on B16 tumor cells, resulting in NF-κB activation, Bcl-2 and Bcl-xL upregulation, and Bax downregulation, which finally leads to tumor cell resistance to apoptosis. They also reported that Tim-3 facilitates the survival of B16 cells in the blood, increasing the number of metastatic nodules in the lung. Thus, blockade of the Tim-3 pathway may help prevent metastasis. Subsequently, using gene microarray analysis, Huang et al. (38) showed that Tim-3 expression was significantly increased in ECs from patients with lymphoma compared to ECs from controls. They also showed that Tim-3 on ECs contributed to the lymphoma endothelium-facilitated growth and dissemination of lymphoma by interacting with circulating T cells and suppressing the activation of CD4^+^ T cells. Moreover, Tim-3^+^ ECs were found to suppress the activation of CD4^+^ T lymphocytes through activation of the IL-6-STAT3 pathway and to inhibit Th1 polarization, leading to tumor immune evasion. Their data suggest that tissue cells expressing Tim-3 can also negatively regulate the immune response by inhibiting the activity of T cells. Their findings highlight a novel role of Tim-3 in tumor immune evasion, providing further support for using Tim-3 as a therapeutic target for tumors.

### Other roles of Tim-3 in innate immunity

γδ T cells, or NKT cells, are considered to be one of the bridges between innate and adaptive immunity. There are a few published reports on the role of Tim-3 expression on γδ T cells or NKT cells. In pregnant women with pre-eclampsia, decreased Tim-3 expression on γδ T cells was found to be associated with increased IFN-γ production by these cells (39), suggesting that Tim-3 limits γδ T cell activation. In addition, a negative correlation has been observed between Tim-3 expression and TNF production by NKT cells (40), suggesting a suppressive role of Tim-3 in NKT functions.

T cell immunoglobulin and mucin domain protein-3 also actively contributes to the homeostasis of innate immunity in other ways. For example, Jayaraman et al. (41) showed that the binding of Tim-3-Ig to cell-surface Gal-9, expressed by infected macrophages, activated an antimicrobial program that restricts the intracellular replication of *M. tuberculosis*. They further showed that the Tim-3-Gal-9 interaction promoted macrophage clearance of intracellular pathogens by inducing caspase-1-dependent IL-1β secretion. In another report (42), Tim-3 was found to be involved in the expansion of myeloid-derived suppressor cells (MDSCs). Heterogeneous CD11b^+^Gr-1^+^ myeloid cells, which expand in tumor-bearing individuals and in the presence of infection, are potent suppressors of T cell responses (43). Dardalhon et al. (42) reported that overexpression of Gal-9 under the control of the actin promoter resulted in an increase in CD11b^+^Ly6G^+^ cells and inhibition of immune responses. Additionally, knock out of Tim-3 reduced numbers of CD11b^+^Ly6G^+^ cells, while transgenic mice overexpressing Tim-3 showed accelerated growth of EL4 lymphoma and an increased frequency of MDSCs in the spleen compared to wild-type mice. Ji et al. (44) demonstrated that HCV-infected human hepatocytes showed increased Tim-3 expression and, when co-cultured with human CD4^+^ T cells, drove their conversion to CD25^+^Foxp3^+^ Treg cells. These data demonstrate that binding of Gal-9 to Tim-3 can also inhibit immune responses by regulating the expansion of immune suppressor cells.

**Figure 2:**
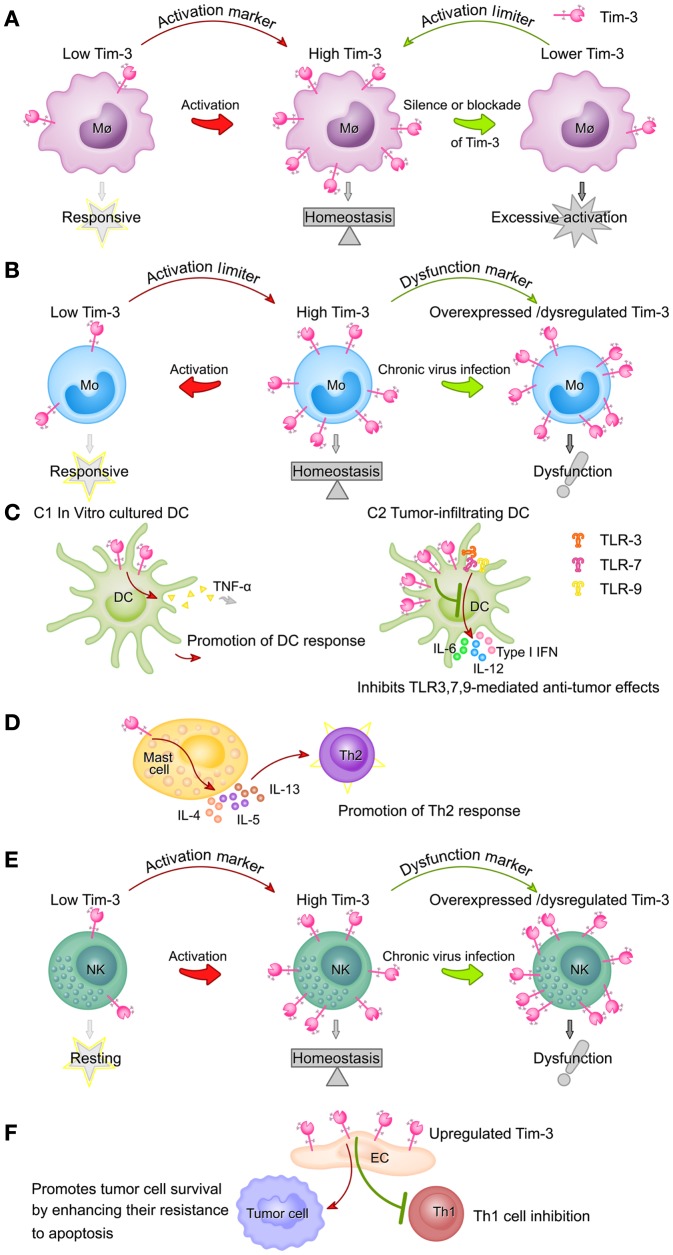
**Summary of Tim-3 expression on different innate immune cell populations and its functions**. **(A)** Tim-3 acts as an activation marker of macrophages and a suppressor of macrophage activity. Tim-3 expression on macrophages is upregulated following activation (e.g., by LPS). High Tim-3 expression contributes to the homeostasis of macrophages, as blockade of the Tim-3 pathway leads to excessive macrophage activation. **(B)** Tim-3 acts as both an activation marker of monocytes and a suppressor of monocyte activity. Tim-3 is constitutively expressed on monocytes at high levels. Tim-3 expression decreases rapidly following stimulation of TLRs and this decrease correlates with increased monocyte activation. However, during chronic viral infection, Tim-3 is overexpressed on monocytes or cannot be downregulated, leading to the dysfunction or exhaustion of monocytes. **(C)** The roles of Tim-3 in dendritic cells are context-dependent. In *in vitro* cultured DCs, Tim-3 signaling promotes DC responses by increasing TNF-α production (C1), while, in tumor-infiltrating DCs, it inhibits the TLR3-, TLR7-, and TLR9-mediated anti-tumor effects of DCs (C2). **(D)** Tim-3 signaling in mast cells promotes Th2 responses by increasing IL-4, IL-5, and IL-13 production. **(E)** Expression of Tim-3 on NK cells is increased following activation and these increased Tim-3 levels act as a marker of fully mature and functional NK cells, but also potentially restrain NK cell function. During chronic viral infection, Tim-3 is overexpressed on NK cells or cannot be downregulated, both of which compromise NK cell function. **(F)** Tim-3 expression on the vascular endothelium is increased in the tumor microenvironment of lymphoma and melanoma, resulting in inhibition of Th1 cell function and increased tumor metastasis.

Tim-3 expression on different innate immune cells and its functions are summarized in Figure 2.

## Concluding Remarks and Future Perspectives

From the evidence presented in this article, it is clear that Tim-3 contributes to immune homeostasis by maintaining optimal activation of innate immune cells. Due to the association between dysregulated Tim-3 expression on innate immune cells and the pathogenesis of tumors and chronic viral infections, it is important to determine the detailed mechanisms regulating Tim-3 expression on different innate immune cells. In addition, although it is known that Tim-3 signaling negatively regulates the activation of innate immune cells, in most cases, the nature of the downstream signaling pathways in the different cell types is not known. The available evidence suggests that Tim-3 may be an attractive target for clinical intervention, and addressing the remaining questions should be a major focus of future studies.

## Conflict of Interest Statement

The authors declare that the research was conducted in the absence of any commercial or financial relationships that could be construed as a potential conflict of interest.

## Abbreviations

DC, dendritic cells; Gal-9, galectin-9; MDSC, myeloid-derived suppressor cells; Tim-3, T cell immunoglobulin and mucin domain protein 3
